# Phenyl naphthalene-2-sulfonate

**DOI:** 10.1107/S1600536808035824

**Published:** 2008-11-08

**Authors:** Jasmine P. Vennila, Helen P. Kavitha, D. John Thiruvadigal, V. Manivannan

**Affiliations:** a Department of Physics, Panimalar Institute of Technology, Chennai 600 095, India; b Department of Chemistry, SRM University, Ramapuram, Chennai 600 089, India; c Department of Physics, SRM University, Kattankulathur Campus, Chennai 603 203, India; d Department of Physics, Presidency College, Chennai 600 005, India

## Abstract

In the crystal structure of the title compound, C_16_H_12_O_3_S, the dihedral angle between the naphthalene ring system and the phenyl ring is 65.21 (3)°. The mol­ecules are linked by inter­molecular C—H⋯O hydrogen bonds, forming a chain along the *a* axis. The chains are connected through weak C—H⋯π inter­actions.

## Related literature

For general background, see: Spungin *et al.* (1984 ▶); Yachi *et al.* (1989 ▶). For related structures, see: Manivannan *et al.* (2005 ▶); Ramachandran *et al.* (2007 ▶).
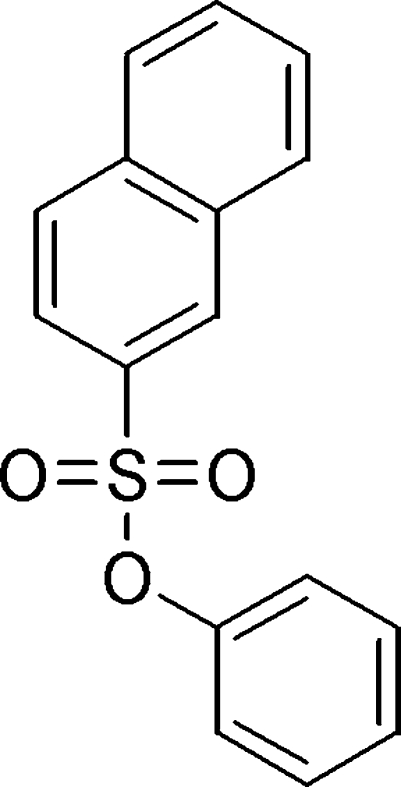


## Experimental

### 

#### Crystal data

C_16_H_12_O_3_S
*M* *_r_* = 284.32
Orthorhombic, 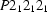

*a* = 6.1525 (2) Å
*b* = 12.7466 (7) Å
*c* = 17.3414 (10) Å
*V* = 1359.97 (12) Å^3^
*Z* = 4
Mo *K*α radiation
μ = 0.24 mm^−1^
*T* = 295 (2) K
0.25 × 0.18 × 0.16 mm

#### Data collection

Bruker Kappa APEXII diffractometer
Absorption correction: multi-scan (**SADABS**; Sheldrick, 1996 ▶) *T*_min_ = 0.942, *T*_max_ = 0.962
11868 measured reflections
5093 independent reflections
3412 reflections with *I* > 2σ(*I*)
*R*_int_ = 0.026

#### Refinement

*R*[*F* ^2^ > 2σ(*F* ^2^)] = 0.044
*wR*(*F* ^2^) = 0.106
*S* = 1.01
5093 reflections
181 parameters
H-atom parameters constrained
Δρ_max_ = 0.26 e Å^−3^
Δρ_min_ = −0.31 e Å^−3^
Absolute structure: Flack (1983 ▶), 1993 Friedel pairs
Flack parameter: −0.03 (7)

### 

Data collection: *APEX2* (Bruker, 2004 ▶); cell refinement: *APEX2* and *SAINT* (Bruker, 2004 ▶); data reduction: *SAINT*; program(s) used to solve structure: *SHELXS97* (Sheldrick, 2008 ▶); program(s) used to refine structure: *SHELXL97* (Sheldrick, 2008 ▶); molecular graphics: *PLATON* (Spek, 2003 ▶); software used to prepare material for publication: *SHELXL97*.

## Supplementary Material

Crystal structure: contains datablocks global, I. DOI: 10.1107/S1600536808035824/is2352sup1.cif

Structure factors: contains datablocks I. DOI: 10.1107/S1600536808035824/is2352Isup2.hkl

Additional supplementary materials:  crystallographic information; 3D view; checkCIF report

## Figures and Tables

**Table 1:** Hydrogen-bond geometry (Å, °)

*D*—H⋯*A*	*D*—H	H⋯*A*	*D*⋯*A*	*D*—H⋯*A*
C2—H2⋯O2^i^	0.93	2.51	3.424 (2)	169
C5—H5⋯*Cg*2^ii^	0.93	2.96	3.486 (2)	117
C6—H6⋯*Cg*3^ii^	0.93	2.94	3.535 (2)	123
C12—H12⋯*Cg*1^iii^	0.93	2.94	3.788 (3)	152

Symmetry codes: (i) 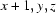; (ii) 
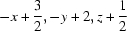
; (iii) 
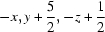
. *Cg*1 is the centroid of the C1–C6 ring, *Cg*2 is the centroid of the C7–C9/C14–C16 ring and *C*g3 is the centroid of the C9–C14 ring.

**Fig. 1.**
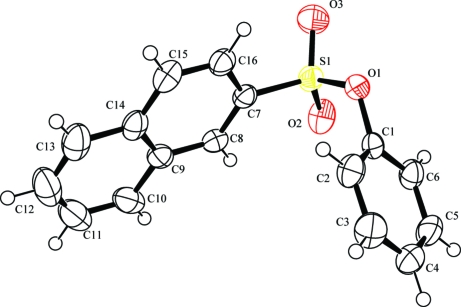
The molecular structure of (I), with atom labels and 50% probability displacement ellipsoids for non-H atoms.

**Fig. 2.**
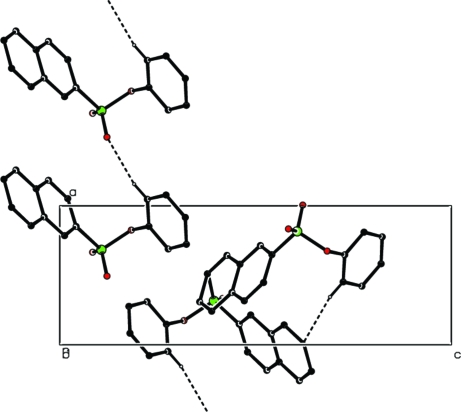
The packing of (I), viewed down the b axis. Hydrogen bonds are shown as dashed lines. H atoms not involved in hydrogen bonding have been omitted for the sake of clarity.

## supplementary crystallographic information

### Comment

Several compounds containing the *para* -toluene sulfonate moiety are used in the fields of biology and industry. The merging of lipids can be monitored using a derivative of *para*-toluene sulfonate (Yachi *et al.*, 1989). This method has been used in studying the membrane fusion during the acrosome reaction (Spungin *et al.*, 1984).

The geometric parameters in the title compound (I) agree well with the reported values of similar structures (Manivannan *et al.*, 2005; Ramachandran *et al.*, 2007). The phenyl ring makes a dihedral angle of 65.21 (3)° with the naphthalene ring system. The torsion angles of O2—S1—C7—C8 and O3—S1—C7—C16 [2.04 (15)° and 46.94 (15)°, respectively] indicate the *syn* conformation of the sulfonyl moiety. The crystal structure is stabilized through weak intermolecular C—H···O and C—H···π interactions (Fig. 2 and Table 1). Cg1, Cg2 and Cg3 are the centroids of the C1–C6 ring, the C7–C9/C14–C16 ring and the C9–C14 ring, respectively.

### Experimental

2-Naphthalene sulfonyl chloride (2.5 mmol) dissolved in acetone (4 ml) was added dropwise to phenol (2.5 mmol) in aqueous NaOH solution (4 ml, 5%) with constant shaking. The precipitated compound (2 mmol, yield 77%) recrystallized from ethanol yielded colourless crystals.

### Refinement

H atoms were positioned geometrically and refined using riding model, with C—H = 0.93 Å and *U*_iso_(H) = 1.2*U*_eq_(C).

### Crystal data

**Table:**

C_16_H_12_O_3_S	*F*_000_ = 592
*M**_r_* = 284.32	*D*_x_ = 1.389 Mg m^−^^3^
Orthorhombic, *P*2_1_2_1_2_1_	Mo *K*α radiation λ = 0.71073 Å
Hall symbol: P 2ac 2ab	Cell parameters from 4818 reflections
*a* = 6.1525 (2) Å	θ = 2.2–25.4º
*b* = 12.7466 (7) Å	µ = 0.24 mm^−^^1^
*c* = 17.3414 (10) Å	*T* = 295 (2) K
*V* = 1359.97 (12) Å^3^	Tablet, colourless
*Z* = 4	0.25 × 0.18 × 0.16 mm

### Data collection

**Table:**

Bruker Kappa APEXII diffractometer	5093 independent reflections
Radiation source: fine-focus sealed tube	3412 reflections with *I* > 2σ(*I*)
Monochromator: graphite	*R*_int_ = 0.027
*T* = 295(2) K	θ_max_ = 34.9º
ω and φ scans	θ_min_ = 2.4º
Absorption correction: multi-scan(*SADABS*; Sheldrick, 1996)	*h* = −9→5
*T*_min_ = 0.942, *T*_max_ = 0.962	*k* = −19→18
11868 measured reflections	*l* = −25→27

### Refinement

**Table:**

Refinement on *F*^2^	Hydrogen site location: inferred from neighbouring sites
Least-squares matrix: full	H-atom parameters constrained
*R*[*F*^2^ > 2σ(*F*^2^)] = 0.044	*w* = 1/[σ^2^(*F*_o_^2^) + (0.0476*P*)^2^ + 0.0596*P*] where *P* = (*F*_o_^2^ + 2*F*_c_^2^)/3
*wR*(*F*^2^) = 0.106	(Δ/σ)_max_ < 0.001
*S* = 1.01	Δρ_max_ = 0.26 e Å^−^^3^
5093 reflections	Δρ_min_ = −0.31 e Å^−^^3^
181 parameters	Extinction correction: none
Primary atom site location: structure-invariant direct methods	Absolute structure: Flack (1983), 1993 Friedel pairs
Secondary atom site location: difference Fourier map	Flack parameter: −0.03 (7)

### Fractional atomic coordinates and isotropic or equivalent isotropic displacement parameters (Å^2^)

**Table:**

	*x*	*y*	*z*	*U*_iso_*/*U*_eq_	
S1	0.68361 (7)	0.89579 (3)	0.10630 (3)	0.03997 (11)	
O1	0.8255 (2)	0.88973 (9)	0.18279 (6)	0.0403 (3)	
O2	0.4945 (2)	0.95689 (12)	0.12058 (8)	0.0527 (4)	
O3	0.6661 (3)	0.78918 (10)	0.08397 (9)	0.0646 (4)	
C1	0.8523 (3)	0.98154 (12)	0.22766 (9)	0.0332 (3)	
C2	1.0472 (3)	1.03331 (16)	0.22378 (11)	0.0438 (4)	
H2	1.1557	1.0114	0.1901	0.053*	
C3	1.0781 (3)	1.11934 (17)	0.27152 (13)	0.0537 (5)	
H3	1.2089	1.1558	0.2701	0.064*	
C4	0.9168 (3)	1.15065 (16)	0.32060 (12)	0.0513 (5)	
H4	0.9389	1.2084	0.3524	0.062*	
C5	0.7234 (3)	1.09789 (16)	0.32342 (10)	0.0497 (5)	
H5	0.6144	1.1203	0.3567	0.060*	
C6	0.6895 (3)	1.01146 (14)	0.27702 (10)	0.0422 (4)	
H6	0.5594	0.9744	0.2792	0.051*	
C7	0.8516 (3)	0.96307 (12)	0.04171 (9)	0.0334 (3)	
C8	0.7855 (3)	1.05729 (12)	0.01241 (9)	0.0355 (4)	
H8	0.6522	1.0855	0.0269	0.043*	
C9	0.9203 (3)	1.11168 (13)	−0.03991 (10)	0.0380 (4)	
C10	0.8568 (4)	1.20865 (14)	−0.07297 (12)	0.0522 (5)	
H10	0.7231	1.2379	−0.0601	0.063*	
C11	0.9892 (5)	1.25888 (17)	−0.12299 (12)	0.0652 (6)	
H11	0.9458	1.3223	−0.1446	0.078*	
C12	1.1911 (5)	1.2161 (2)	−0.14254 (12)	0.0709 (7)	
H12	1.2808	1.2516	−0.1769	0.085*	
C13	1.2580 (3)	1.12340 (18)	−0.11191 (12)	0.0569 (5)	
H13	1.3933	1.0963	−0.1251	0.068*	
C14	1.1230 (3)	1.06801 (14)	−0.06014 (10)	0.0405 (4)	
C15	1.1837 (3)	0.96940 (15)	−0.02884 (10)	0.0439 (4)	
H15	1.3164	0.9399	−0.0425	0.053*	
C16	1.0520 (3)	0.91760 (13)	0.02050 (10)	0.0395 (4)	
H16	1.0929	0.8527	0.0403	0.047*	

### Atomic displacement parameters (Å^2^)

**Table:**

	*U*^11^	*U*^22^	*U*^33^	*U*^12^	*U*^13^	*U*^23^
S1	0.0443 (2)	0.0406 (2)	0.0350 (2)	−0.00821 (18)	0.00007 (19)	−0.00279 (17)
O1	0.0525 (7)	0.0344 (5)	0.0339 (6)	0.0047 (6)	−0.0012 (5)	0.0021 (5)
O2	0.0361 (7)	0.0768 (9)	0.0451 (8)	−0.0027 (6)	0.0010 (6)	0.0017 (7)
O3	0.0897 (11)	0.0457 (7)	0.0584 (9)	−0.0245 (7)	0.0029 (9)	−0.0083 (6)
C1	0.0381 (10)	0.0353 (7)	0.0262 (7)	0.0012 (6)	−0.0017 (6)	0.0046 (6)
C2	0.0352 (10)	0.0579 (11)	0.0382 (9)	0.0016 (8)	0.0018 (7)	−0.0005 (8)
C3	0.0463 (12)	0.0649 (13)	0.0498 (12)	−0.0151 (10)	−0.0042 (9)	−0.0019 (10)
C4	0.0665 (14)	0.0508 (11)	0.0365 (11)	−0.0090 (9)	−0.0024 (9)	−0.0056 (8)
C5	0.0563 (13)	0.0562 (11)	0.0366 (9)	−0.0022 (9)	0.0132 (8)	−0.0060 (9)
C6	0.0413 (10)	0.0503 (9)	0.0348 (8)	−0.0094 (8)	0.0053 (8)	0.0016 (7)
C7	0.0373 (9)	0.0340 (7)	0.0290 (7)	−0.0004 (6)	−0.0011 (6)	−0.0049 (6)
C8	0.0374 (9)	0.0343 (7)	0.0348 (8)	0.0011 (7)	−0.0017 (7)	−0.0063 (6)
C9	0.0478 (10)	0.0338 (8)	0.0325 (8)	−0.0053 (7)	−0.0064 (7)	−0.0034 (7)
C10	0.0675 (14)	0.0375 (9)	0.0516 (11)	−0.0044 (8)	−0.0138 (10)	0.0012 (8)
C11	0.0953 (18)	0.0482 (11)	0.0521 (14)	−0.0225 (12)	−0.0208 (13)	0.0102 (9)
C12	0.0927 (18)	0.0760 (15)	0.0440 (12)	−0.0440 (15)	−0.0050 (13)	0.0084 (11)
C13	0.0562 (12)	0.0748 (13)	0.0396 (10)	−0.0233 (10)	0.0020 (8)	−0.0062 (10)
C14	0.0419 (11)	0.0515 (10)	0.0280 (8)	−0.0102 (8)	−0.0035 (7)	−0.0074 (7)
C15	0.0378 (10)	0.0594 (10)	0.0347 (9)	0.0062 (9)	−0.0008 (8)	−0.0070 (8)
C16	0.0451 (10)	0.0425 (9)	0.0310 (9)	0.0101 (7)	−0.0023 (7)	−0.0024 (7)

### Geometric parameters (Å, °)

**Table:**

S1—O3	1.4172 (13)	C7—C16	1.411 (2)
S1—O2	1.4217 (15)	C8—C9	1.411 (2)
S1—O1	1.5899 (13)	C8—H8	0.9300
S1—C7	1.7487 (17)	C9—C14	1.410 (3)
O1—C1	1.4149 (19)	C9—C10	1.417 (2)
C1—C2	1.370 (2)	C10—C11	1.351 (3)
C1—C6	1.372 (2)	C10—H10	0.9300
C2—C3	1.387 (3)	C11—C12	1.398 (4)
C2—H2	0.9300	C11—H11	0.9300
C3—C4	1.367 (3)	C12—C13	1.360 (3)
C3—H3	0.9300	C12—H12	0.9300
C4—C5	1.368 (3)	C13—C14	1.412 (3)
C4—H4	0.9300	C13—H13	0.9300
C5—C6	1.380 (3)	C14—C15	1.419 (3)
C5—H5	0.9300	C15—C16	1.351 (3)
C6—H6	0.9300	C15—H15	0.9300
C7—C8	1.366 (2)	C16—H16	0.9300
			
O3—S1—O2	120.72 (10)	C7—C8—C9	119.74 (16)
O3—S1—O1	102.89 (9)	C7—C8—H8	120.1
O2—S1—O1	109.31 (7)	C9—C8—H8	120.1
O3—S1—C7	109.88 (9)	C14—C9—C8	119.07 (16)
O2—S1—C7	109.05 (8)	C14—C9—C10	119.18 (18)
O1—S1—C7	103.52 (7)	C8—C9—C10	121.74 (17)
C1—O1—S1	118.86 (10)	C11—C10—C9	120.4 (2)
C2—C1—C6	122.41 (16)	C11—C10—H10	119.8
C2—C1—O1	118.25 (15)	C9—C10—H10	119.8
C6—C1—O1	119.20 (15)	C10—C11—C12	120.4 (2)
C1—C2—C3	118.10 (17)	C10—C11—H11	119.8
C1—C2—H2	121.0	C12—C11—H11	119.8
C3—C2—H2	121.0	C13—C12—C11	120.9 (2)
C4—C3—C2	120.22 (18)	C13—C12—H12	119.6
C4—C3—H3	119.9	C11—C12—H12	119.6
C2—C3—H3	119.9	C12—C13—C14	120.3 (2)
C3—C4—C5	120.68 (18)	C12—C13—H13	119.8
C3—C4—H4	119.7	C14—C13—H13	119.8
C5—C4—H4	119.7	C9—C14—C13	118.75 (19)
C4—C5—C6	120.22 (17)	C9—C14—C15	119.15 (17)
C4—C5—H5	119.9	C13—C14—C15	122.09 (19)
C6—C5—H5	119.9	C16—C15—C14	121.17 (18)
C1—C6—C5	118.37 (17)	C16—C15—H15	119.4
C1—C6—H6	120.8	C14—C15—H15	119.4
C5—C6—H6	120.8	C15—C16—C7	119.23 (16)
C8—C7—C16	121.62 (16)	C15—C16—H16	120.4
C8—C7—S1	119.58 (13)	C7—C16—H16	120.4
C16—C7—S1	118.80 (12)		
			
O3—S1—O1—C1	−171.58 (12)	S1—C7—C8—C9	−179.81 (12)
O2—S1—O1—C1	−42.13 (14)	C7—C8—C9—C14	−0.9 (2)
C7—S1—O1—C1	73.97 (13)	C7—C8—C9—C10	178.76 (16)
S1—O1—C1—C2	−104.08 (16)	C14—C9—C10—C11	−0.3 (3)
S1—O1—C1—C6	80.26 (17)	C8—C9—C10—C11	−179.92 (18)
C6—C1—C2—C3	−0.4 (3)	C9—C10—C11—C12	−0.3 (3)
O1—C1—C2—C3	−175.92 (16)	C10—C11—C12—C13	0.2 (3)
C1—C2—C3—C4	−0.1 (3)	C11—C12—C13—C14	0.6 (3)
C2—C3—C4—C5	0.0 (3)	C8—C9—C14—C13	−179.35 (16)
C3—C4—C5—C6	0.6 (3)	C10—C9—C14—C13	1.0 (2)
C2—C1—C6—C5	1.0 (3)	C8—C9—C14—C15	1.5 (2)
O1—C1—C6—C5	176.47 (15)	C10—C9—C14—C15	−178.11 (16)
C4—C5—C6—C1	−1.1 (3)	C12—C13—C14—C9	−1.2 (3)
O3—S1—C7—C8	132.35 (14)	C12—C13—C14—C15	177.93 (18)
O2—S1—C7—C8	−2.04 (15)	C9—C14—C15—C16	−0.8 (3)
O1—S1—C7—C8	−118.32 (13)	C13—C14—C15—C16	−179.89 (18)
O3—S1—C7—C16	−46.94 (15)	C14—C15—C16—C7	−0.6 (3)
O2—S1—C7—C16	178.67 (12)	C8—C7—C16—C15	1.3 (2)
O1—S1—C7—C16	62.39 (13)	S1—C7—C16—C15	−179.43 (14)
C16—C7—C8—C9	−0.5 (2)		

### Hydrogen-bond geometry (Å, °)

**Table:**

*D*—H···*A*	*D*—H	H···*A*	*D*···*A*	*D*—H···*A*
C2—H2···O2^i^	0.93	2.51	3.424 (2)	169
C5—H5···Cg2^ii^	0.93	2.96	3.486 (2)	117
C6—H6···Cg3^ii^	0.93	2.94	3.535 (2)	123
C12—H12···Cg1^iii^	0.93	2.94	3.788 (3)	152

Symmetry codes: (i) *x*+1, *y*, *z*; (ii) −*x*+3/2, −*y*+2, *z*+1/2; (iii) −*x*, *y*+5/2, −*z*+1/2.
